# Effect of Temporary Anchorage Devices on class II anterior open bite malocclusion patient

**DOI:** 10.1002/ccr3.9435

**Published:** 2025-03-07

**Authors:** Justin Nguyen, Chris Cramer, Steven Park, Hayden Jones, Ivan Alpizar, Man Hung

**Affiliations:** ^1^ College of Dental Medicine Roseman University of Health Sciences South Jordan Utah USA; ^2^ Cramer Orthodontics Denton Texas USA; ^3^ Department of Orthopaedic Surgery Operations University of Utah Salt Lake City Utah USA

**Keywords:** anterior open bite, Class II malocclusion, dental, nonsurgical treatment, orthodontics, TAD

## Abstract

**Key Clinical Message:**

Temporary Anchorage Devices‐supported molar intrusion can be an effective non‐surgical alternative for treating Class II anterior open bite in skeletally mature patients, resulting in an improved occlusal relationship and enhanced chin projection through forward autorotation of the mandible.

**Abstract:**

Temporary Anchorage Devices are titanium alloy miniscrews utilized to provide maximum orthodontic anchorage during the correction of various malocclusions. This case report examined the use of TADs to support posterior intrusion while correcting a patient's Class II anterior open bite malocclusion in support of the patient's request to avoid surgical intervention.

## 1 INTRODUCTION

Class II malocclusion is a common occurrence, reportedly affecting 15% of the population in the US.^1^ Orthodontic treatment of these cases can be particularly challenging, especially in non‐growing patients. Depending on the patient's age, skeletal discrepancy, and Class II severity, treatment options range from less invasive techniques such as removable appliances to more invasive options including premolar extraction. In the most severe cases, orthognathic surgery is usually required.^1^, ^2^, ^3^, ^4^ An enhanced technique for increasing the range of issues that can be addressed non‐surgically is the use of Temporary Anchorage Devices (TADs), which are proving highly effective in correcting Class II malocclusions.^5^, ^6^, ^7^ TADs are titanium alloy miniscrews which provide localized skeletal anchorage points to achieve desired orthodontic tooth movements of specific teeth while minimizing undesired reciprocal forces on adjacent teeth. This allows more predictable and comprehensive occlusal correction of more severe cases than was previously possible with orthodontic mechanics alone.

In this case report, TAD‐supported molar intrusion in conjunction with orthodontic therapy improved a patient's molar relationship from class II to class I while also closing her anterior open bite and improving her facial profile through autorotation of the mandible.

## 2 CASE PRESENTATION

A 21‐year‐old female patient presented with a chief complaint of “only my back teeth touch” due to her skeletal anterior open bite. Although she underwent orthodontic therapy as an adolescent and achieved good alignment, she was informed orthognathic surgery would be required following growth cessation in order to obtain a full occlusal correction. Now a young adult, the patient did not want to undergo orthognathic surgery and sought alternative treatment options to improve her chewing function.

**FIGURE 1:**
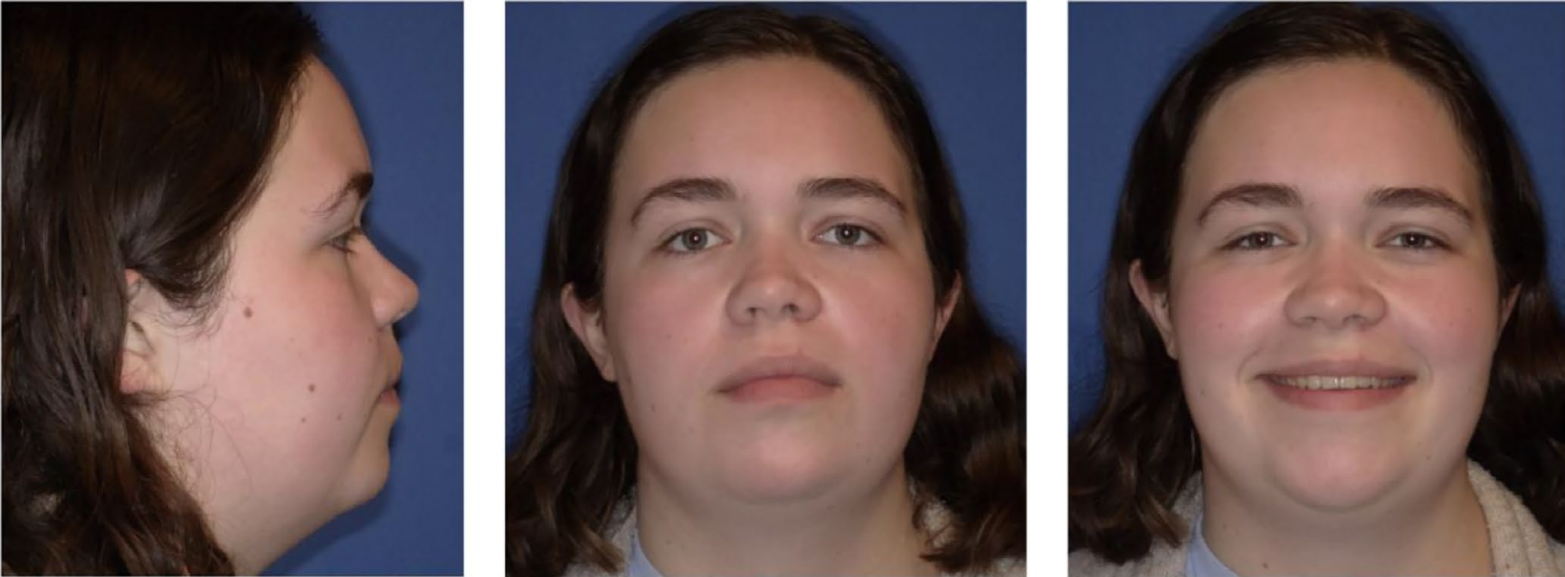
Pre‐treatment extraoral photos show a convex facial profile and a long lower third facial height.

**FIGURE 2:**
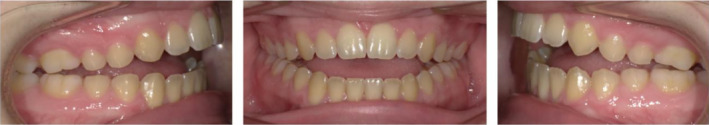
Pre‐treatment intraoral photos show good orthodontic alignment, a 1/2 step class II molar relationship, and a 6 mm anterior open bite.

**FIGURE 3:**
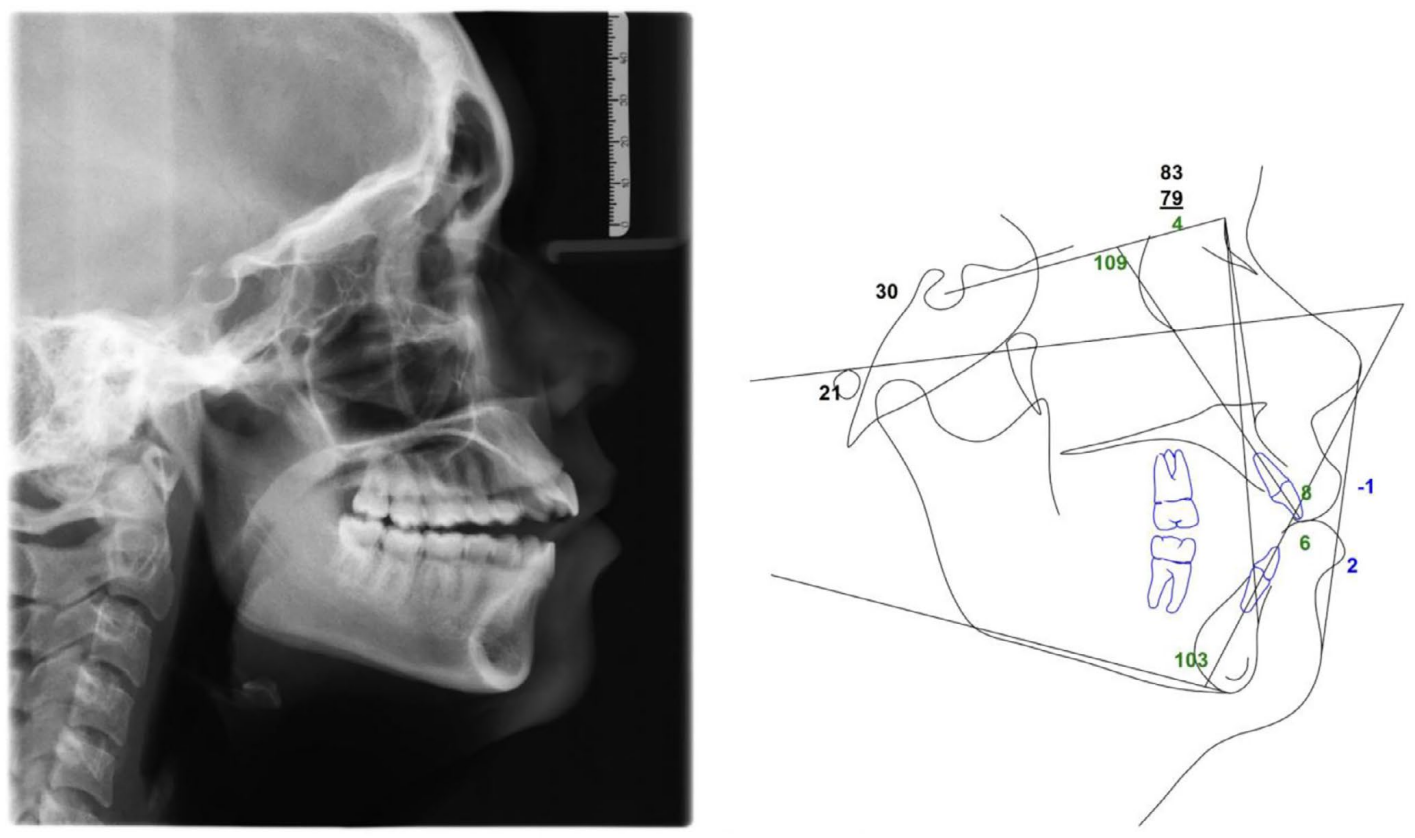
Pre‐treatment cephalometric radiograph and tracing.

Clinically, the patient presented with a convex facial profile and long lower third facial height (Figure 1). The patient had acceptable orthodontic alignment, a half‐step class II molar relationship, and an anterior open bite of approximately 6 mm (Figure 2). The cephalometric tracing showed her mandibular plane angle (MPA) was within normal limits (MPA 30), but it also revealed proclined upper and lower incisors (U1‐SN 109, IMPA 103), protrusive upper and lower lips (UL‐E‐1, LL‐E 2), and a class II skeletal relationship (ANB 4) (Figure 3).

**FIGURE 4:**
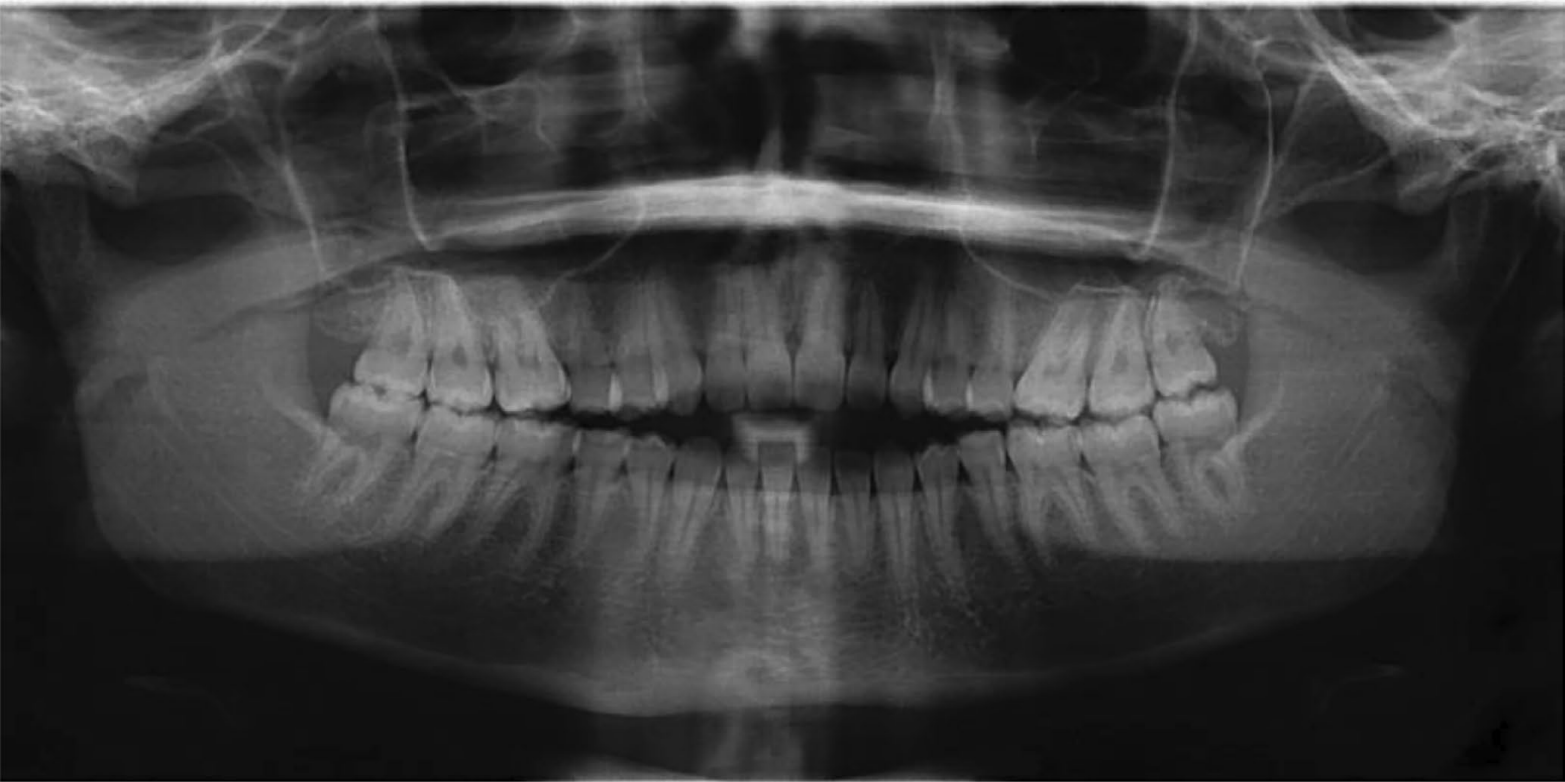
Pre‐treatment panoramic radiograph shows third molars are present.

The patient's temporomandibular joint (TMJ) was evaluated during the initial exam and found to be within normal limits with adequate maximum opening and excursive movements, no joint clicking or popping, and no pain reported by the patient. The patient's third molars were present on the pano, but asymptomatic. (Figure 4).

## 3 TREATMENT PLAN

### 3.1 Treatment objective

The treatment objective was to close the patient's anterior open bite and improve her occlusal function. When the patient denied surgery, the option and limitations of non‐surgical, orthodontic‐only treatment with TADs to provide skeletal anchorage for molar intrusion were explained. The patient elected the non‐surgical option in lieu of surgery. In order to correct the class II dental and skeletal relationship and close the anterior open bite, the molars would be intruded by attaching power thread between each TAD and the respective posterior portions of the upper and lower wires thereby allowing the mandible to rotate forward into an improved molar relationship.

### 3.2 Treatment progress

**FIGURE 5:**
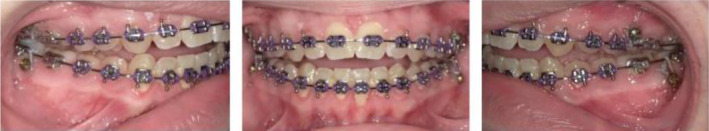
Progress records at 3 months illustrate TAD placement with power thread to each posterior segment and anterior open bite reduced by approximately 3 mm.

Treatment began with placement of one 2.0 mm diameter by 10 mm long TAD into each posterior segment and as well as third molar extractions by an oral surgeon. Due to interradicular space constraints, the upper left TAD was placed between the upper left second premolar and first molar while the remaining TADs were all placed between the first and second molars. (Figure 5).

Two weeks later, traditional twin brackets (0.22 slot, CCO prescription) were placed with upper and lower 016 nickel titanium wires. No intrusion forces were applied at this time.

Approximately 8 weeks later, 20 × 20 nickel titanium wires were placed and power thread was tied between each TAD and its respective posterior portions of the upper and lower wires. (Figure 5) The wires were then gradually increased and power thread replaced approximately every 8 weeks. The final wires were 19 × 25 stainless steel.

**FIGURE 6:**
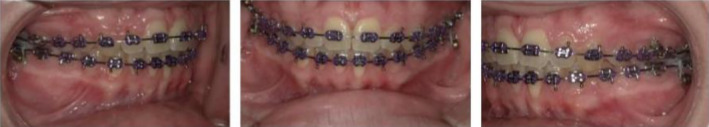
Adequate overbite was achieved within 7 months utilizing TAD anchorage in conjunction with traditional orthodontic therapy.

At approximately 3 months into treatment, the anterior open bite had improved by approximately 3 mm. (Figure 5) At 7 months, the anterior open bite was fully corrected and the patient had about 2 mm of overbite. (Figure 6) The TADs were left in place in case of later relapse so that intrusion forces could be reintroduced if needed, though further molar intrusion was never required.

Finishing elastics were utilized for about 6 months to finalize the anterior bite relationship and included triangle pattern elastics between the upper canines and lower canines and first premolars as well as anterior box elastics between the upper lateral incisors and lower canines. Finishing bends included intrusion steps on the upper and lower second molars and slight extrusion steps in the upper lateral to lateral incisal segment.

**FIGURE 7:**
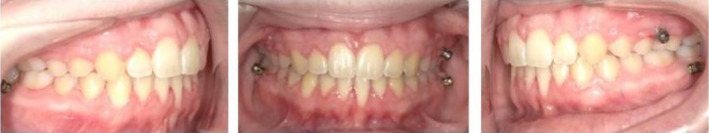
Debond photos show appropriate overbite and Class I molar relationship. The TADs were subsequently removed by the oral surgeon.

At approximately 4 months prior to debond, finishing elastics were discontinued to monitor for anterior open bite relapse potential. No relapse occurred and, at 2 months prior to debond, finishing elastics were resumed to slightly over‐correct the anterior overbite and account for some relapse after appliance removal. The patient was debonded and immediately placed into retention with upper and lower 1 mm thick vacuum formed clear retainers. (Figure 7).

The patient returned to the oral surgeon for TAD removal within 2 weeks of debond. The patient's TMJ remained asymptomatic throughout treatment.

### 3.3 Treatment results

**FIGURE 8:**
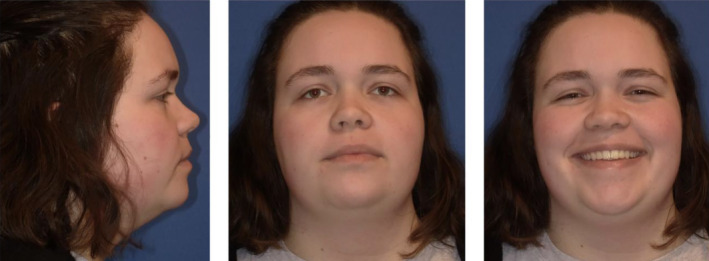
Final facial photos show improved facial profile including reduced lower third facial height, improved chin projection, and improved lip profile.

**FIGURE 9:**
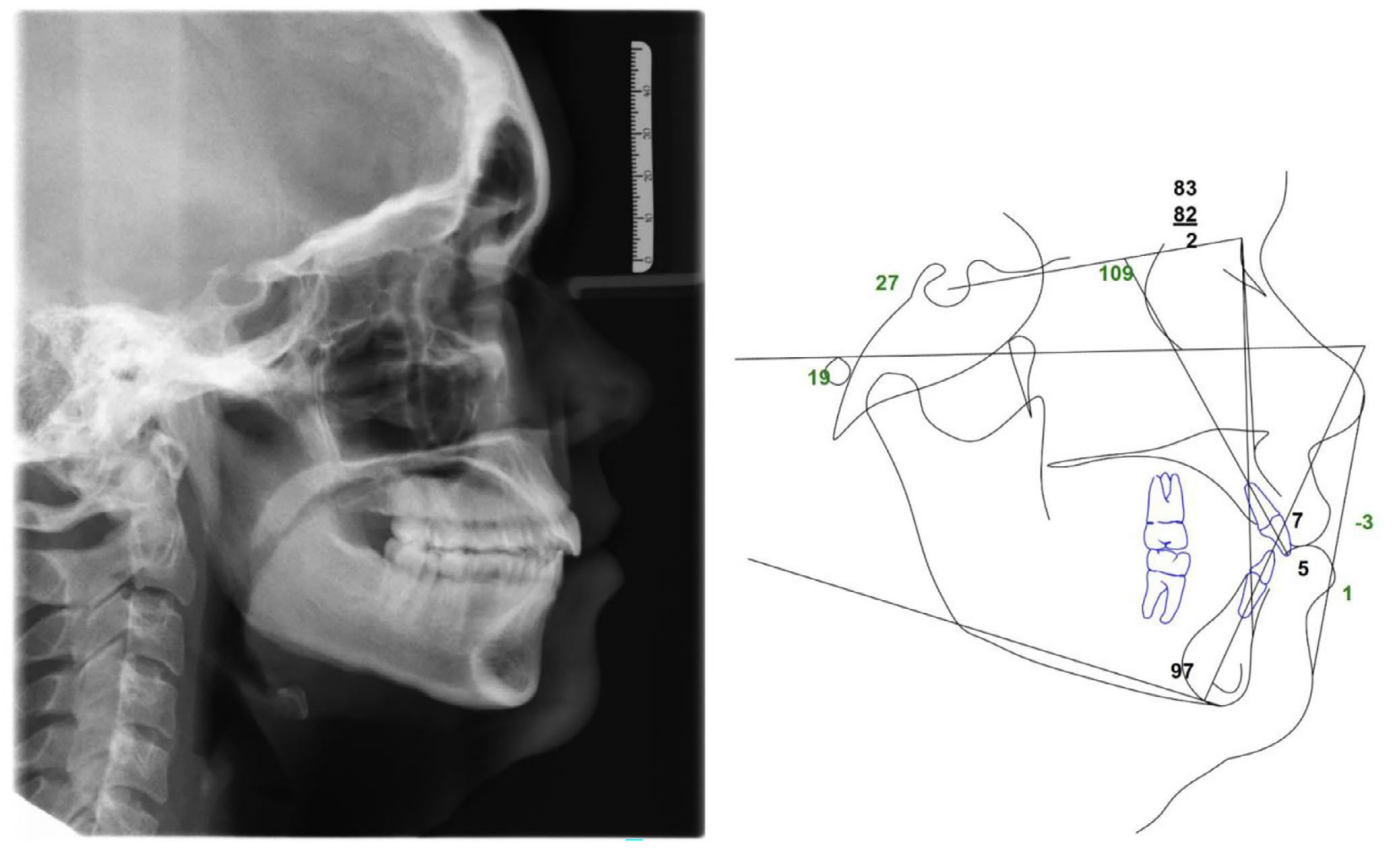
Post‐treatment cephalometric radiograph and tracing show closed anterior open bite and class I occlusion.

The patient achieved a class I molar relationship and a good anterior overbite with an improved facial profile. (Figures 7 and 8) The patient's lateral cephalometric radiograph and tracing show the anterior open bite is now resolved and the occlusion is class I. (Figure 9).

Table 1 summarizes the pre‐ and post‐treatment cephalometric tracing measurements. The skeletal relationship improved to class I (ANB 2). The upper incisor inclination (U1‐SN) remained unchanged while the lower incisor inclination (IMPA) improved by 6°. The upper incisor (U1‐NA) and lower incisor (L1‐NB) procumbency each improved by 1 mm, likely due to B point moving forward. The upper lip procumbency (UL‐E) improved by 2 mm and the lower lip procumbency (LL‐E) improved by 1 mm, likely due to the chin moving forward. The MPA reduced by 3° indicating a forward autorotation.

**TABLE 1:** ABO pre‐ and post‐treatment cephalometric measurements.

ABO cephalometric measurement		Norm	pre‐Tx	post‐Tx
Maxillary position	SNA (Sella nasion to A point)	82°	83°	83°
Mandibular position	SNB (Sella nasion to B point)	80°	79°	81°
Maxillomandibular relationship	ANB (A point to nasion to B point)	2°	4°	2°
Mandibular plane angle	MPA (Sella nasion to Mn plane)	32°	30°	27°
Upper incisor inclination	U1‐SN (Upper incisor to sella nasion)	104°	109°	109°
Lower incisor inclination	IMPA (Lower incisor to Mn plane)	90°	103°	97°
Upper incisor position	U1‐NA (Upper incisor to nasion A point)	4 mm	8 mm	7 mm
Lower incisor position	L1‐NB (Lower incisor to nasion B point)	4 mm	6 mm	5 mm
Upper lip position	UL‐E (Upper lip to E‐line)	−4 mm	−1 mm	−3 mm
Lower lip position	LL‐E (Lower lip to E‐line)	−2 mm	2 mm	1 mm

**FIGURE 10:**
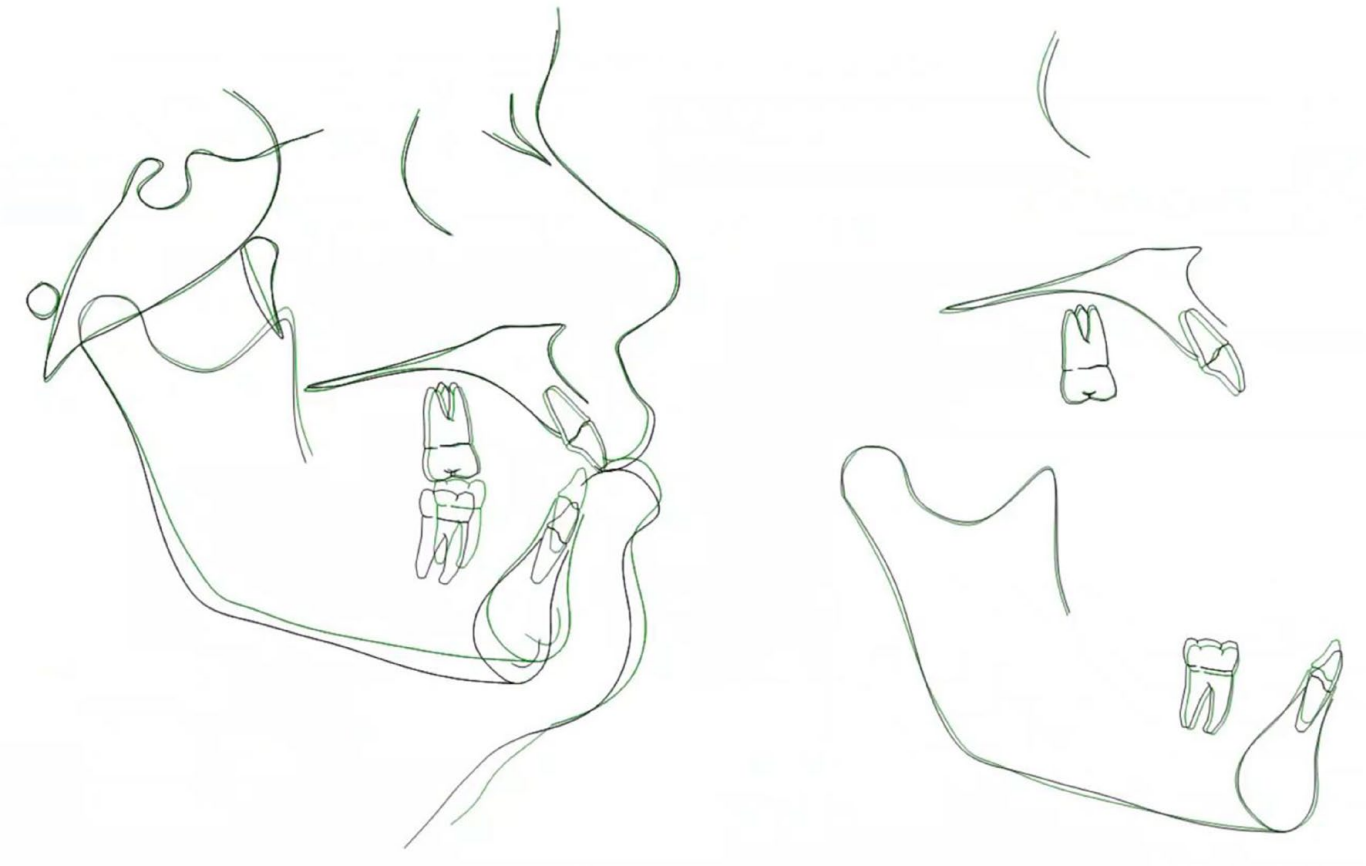
Superimposition of pre‐treatment (black) and post‐treatment (green) cephalometric tracings.

**FIGURE 11:**
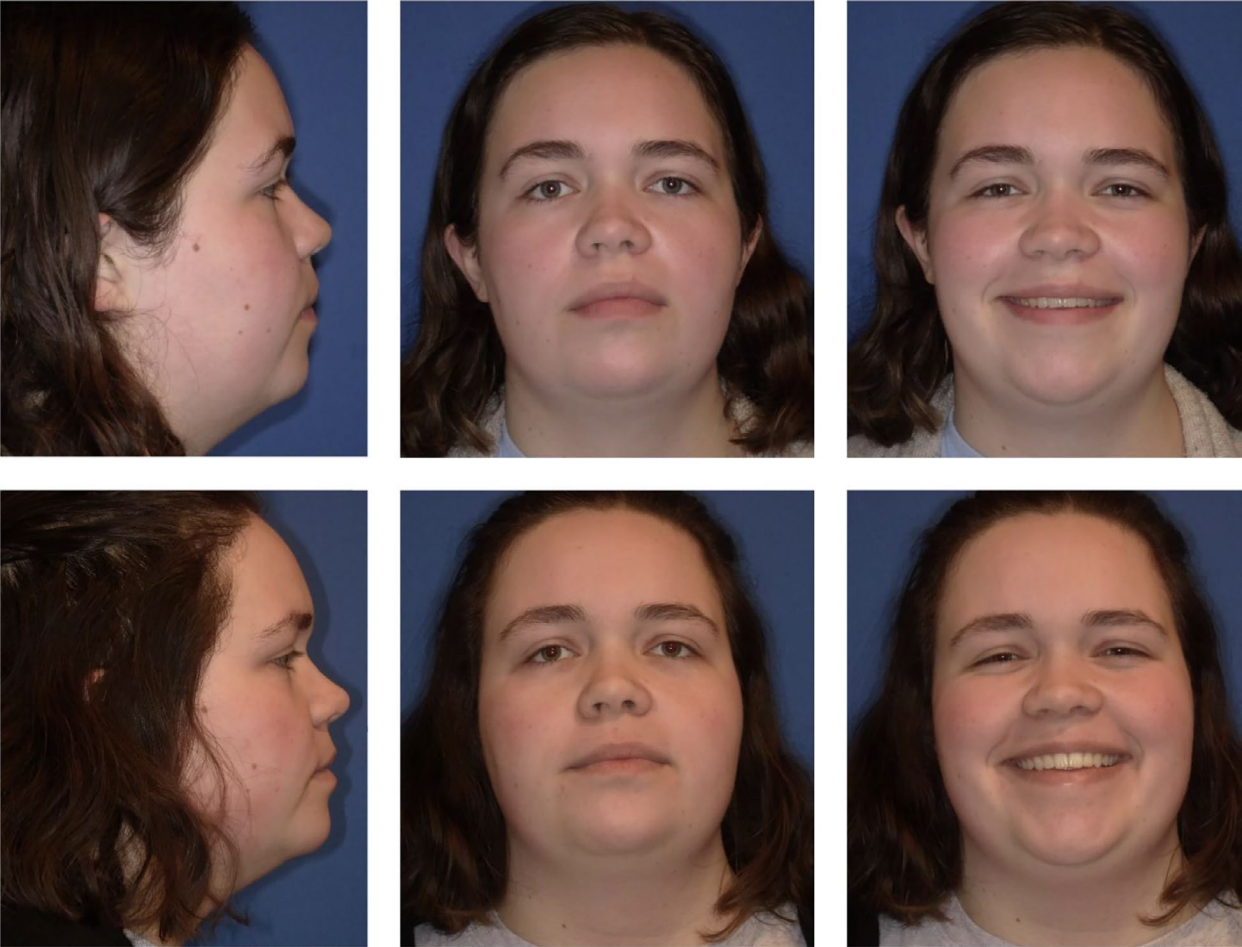
Facial profile photos of initial and debond shows improved convexity and chin projection.

The cephalometric superimposition shows the mandibular molars intruded approximately 2 mm while maxillary molars intruded approximately 1 mm. The superimposition also shows the improved overbite and enhanced chin projection due to the mandibular forward autorotation secondary to molar intrusion. (Figure 10) Facial profile photos also confirm improved facial profile, retracted lips, and enhanced chin projection. (Figure 11).

**FIGURE 12:**
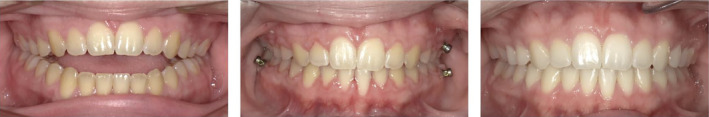
Initial, debond, and 7 months post‐debond demonstrate good open bite correction and post‐treatment stability.

At 7 months, the patient presented for a retainer check and showed good overbite stability (Figure 12).

## 4 DISCUSSION

Class II malocclusion cases present some of the most challenging cases for orthodontic correction, especially after growth cessation. While many class II treatment options exist, treatment of severe class II cases can be limited when the patient denies extractions or orthognathic surgery. To improve outcomes in these more challenging cases, orthodontists often utilize TADs to provide maximum anchorage in order to achieve movements that would otherwise be impossible. Advantages of TAD supported treatment include location versatility due to their small size and reduced need for surgical intervention. This can lead to minimized patient discomfort and lower cost when compared to surgery and improved outcomes when compared to traditional orthodontic only mechanics.^8^ In this case, TADs helped correct a 6 mm anterior open bite by providing skeletal anchorage for molar intrusion which led to forward autorotation of the mandible without incurring orthognathic surgical risks and costs.

Clinicians must evaluate each case carefully to determine if their anterior open bite patient will obtain an overall benefit through TAD supported molar intrusion. Because the mandible auto rotates forward during molar intrusion, the ideal patient begins with a class II occlusal relationship and transitions into a class I. An initial class I molar relationship patient risks becoming class III with reduced overjet or may even finish with traumatic, end‐on anterior occlusion. Alternatively, a class III patient will become a more severe class III with an increased negative overjet.

Also when evaluating options to close an anterior open bite non‐surgically, clinicians may intrude posterior teeth, extrude anterior teeth, or both, though each option must be carefully considered for its respective pros and cons. For patients with an increased lower facial height, posterior intrusion offers the benefit of reducing facial height and reducing convexity as the mandible rotates forward.^9^ For patients with a reduced anterior facial height and minimal incisal display, anterior extrusion may be considered though this is generally considered to be more prone to relapse whereas posterior molar intrusion shows similar stability to orthognathic surgery.^10^

In this case, a non‐growing patient presented with a 6 mm anterior open bite, ½ step class II molar relationship, facial convexity with lip protrusion, and an excess lower ⅓ facial height. In light of her denying orthognathic surgery, posterior intrusion was suggested to help improve her anterior open bite knowing her facial profile could at least tolerate and may even improve as a secondary outcome. Although the patient was thoroughly informed a more ideal skeletal and dental relationship would likely be achieved with surgery and that long term stability may be better when compared with a non‐surgical treatment, the patient understood the risks and benefits and elected the TAD‐supported, orthodontic treatment.

As seen in Table 1, the patient initially had a slightly prognathic maxilla and a slightly retrognathic mandible resulting in a class II skeletal relationship (ANB 4). Also, she had slightly excessive incisal and lip protrusion, but adequate incisal display. Following orthodontic treatment with molar intrusion, her skeletal relationship improved to a class I skeletal relationship (ANB 2) and her dental occlusion improved to a class I molar and canine. It is also interesting to note that her upper incisal position improved slightly (U1‐NA reduced by 1 mm), likely due to the bracket prescription. The lower incisors also became slightly less procumbant (L1‐NB reduced by 1 mm) and less proclined (IMPA reduced by 6°), which could be a combination the bracket prescription and the relative change in B point with the forward mandibular rotation.

While some cephalometric values remain outside of recommended normal ranges, the primary objective was to improve her anterior incisal relationship so that she could chew normally without undergoing jaw surgery. With an improvement from an open bite of 6 mm to an overbite of 2 mm, this objective was satisfied. Additionally, her facial profile and molar relationship improved due to the 3° forward mandibular rotation as demonstrated by the ceph tracings and also seen on the superimposition. (Figure 9).

One downside to treatment with TADs is the possibility of failures and this did occur during this case. The upper left TAD became loose at about 4 months and the patient returned to the surgeon who removed and repositioned the loose TAD. It was allowed to stabilize without intrusion force for about 6 weeks, after which the intrusion force was once again applied. The TAD subsequently remained in place without incident for the remainder of treatment. This patient experienced a 25% TAD failure rate, which is in line with a 2009 meta‐analysis showing a 16.4% TAD failure rate.^11^ Various studies have attempted to isolate the reasons for TAD failures and most agree that sex, age, and which side of the jaw the TADs were placed in are not correlated to the failure rate, while TAD diameter, length, and location are important factors for TAD success.^12^, ^13^, ^14^ TADs of 2.0 mm diameter are more successful than 1.8 mm TADs.^9^ Also, placement in attached gingiva leads to lower failure rates than TADs placed in moveable mucosa.^13^ An interesting research finding is failure rates do not differ between orthodontists and surgeons, though both require sufficient training for a high success rate.^11^

As with any procedure, the patient needs to be informed of the risks of a TAD failure and the need to have it replaced promptly should this occur. Also, the clinician needs to check each TAD for possible failure at each appointment, because the patient may not realize a failure has occurred. In this case, the patient exhibited no other symptom than the TAD was found to be mobile under moderate tactile pressure applied by the clinician. In this instance, the power thread was removed and the patient was referred back to the oral surgeon who managed the failed TAD as stated above.

Another consideration regarding molar intrusion versus orthognathic surgery to close an anterior open bite is long term stability. A 2011 meta‐analysis showed TAD supported molar intrusion to be 75% stable compared to 82% stability in surgical correction of anterior open bites.^10^ However, two more recent studies showed TAD supported molar intrusion produces similar stability.^15^, ^16^ This reduces the previously perceived stability benefit of surgery over TAD supported molar intrusion treatment. This patient demonstrated good stability at the 7 months retainer check. (Figure 12) In any case, the patient needs to understand that some relapse is likely, though the overall improvement can be significant and life changing.

## 5 CONCLUSIONS

TADs have become a crucial component in orthodontic treatment by providing skeletal anchorage to move target teeth while minimizing unwanted movements on other teeth. This allows clinicians to consider more advanced treatments and achieve outcomes that were previously not possible without orthognathic surgery. This case report demonstrates the use of TAD‐supported molar intrusion in conjunction with traditional orthodontic techniques in order to eliminate a significant anterior open bite and correct a class II molar relationship. The skeletal relationship and occlusal improvement resulted from mandibular autorotation with secondary benefits of improving lip protrusion and facial profile.

During the course of treatment, one of four TADs failed highlighting the need to properly inform the patient of the risks associated with TADs. TAD failures are a realistic possibility and the clinician should understand the nuances of TAD selection and placement to reduce the risk of failure. Even in light of this risk, this case demonstrates the possibility of significantly improving a patient's occlusion at a lower overall treatment cost and lower risk compared to orthognathic surgery.

## AUTHOR CONTRIBUTIONS

**Justin Nguyen:** Conceptualization; data curation; formal analysis; visualization; writing – original draft; writing – review and editing. **Chris Cramer:** Conceptualization; data curation; formal analysis; methodology; resources; supervision; validation; visualization; writing – original draft; writing – review and editing. **Steven Park:** Methodology; visualization; writing – original draft; writing – review and editing. **Hayden Jones:** Visualization; writing – original draft; writing – review and editing. **Ivan Alpizar:** Visualization; writing – original draft; writing – review and editing. **Man Hung:** Conceptualization; methodology; project administration; resources; supervision; validation; visualization; writing – original draft; writing – review and editing.

## FUNDING INFORMATION

No external funding was received for this study.

## CONFLICT OF INTEREST STATEMENT

The authors declare no conflicts of interest.

## CONSENT

Written informed consent was obtained from the patient to publish this report in accordance with the journal's patient consent policy.

## Data Availability

The data that support the findings of this study are available in the supplementary material of this article.
